# Effect of Silicon Concentration on the Properties of Al-Cr-Si-N Coatings Deposited Using Cathodic Arc Evaporation

**DOI:** 10.3390/ma13214717

**Published:** 2020-10-22

**Authors:** Bogdan Warcholinski, Adam Gilewicz, Piotr Myslinski, Ewa Dobruchowska, Dawid Murzynski, Tatyana A. Kuznetsova

**Affiliations:** 1 Faculty of Mechanical Engineering, Koszalin University of Technology, Sniadeckich 2, 75-453 Koszalin, Poland; adam.gilewicz@tu.koszalin.pl (A.G.); piotr.myslinski@tu.koszalin.pl (P.M.); ewa.dobruchowska@tu.koszalin.pl (E.D.); dawid.murzynski@tu.koszalin.pl (D.M.); 2 A.V. Luikov Institute of Heat and Mass Transfer of the National Academy of Science of Belarus, vulica Pietrusia Broŭki 15, 220072 Minsk, Belarus; t_kuzn@hmti.ac.by

**Keywords:** AlCrSiN, structure, hardness, adhesion, wear, corrosion

## Abstract

The current market requirements are related to the introduction of new protective coatings for tools and machine parts with much better performance properties. These requirements are met by the AlCrSiN coatings; however, knowledge on the adhesion of these coatings to the substrate, as well as on their corrosion resistance, is deficient. The article presents the results of technological works on the coating deposition from AlCrSi cathodes with a silicon concentration from 0 at% to 10 at% by the cathodic arc evaporation and the results of systematic studies of their structure, mechanical, tribological and electrochemical properties. A correlation between the above-mentioned properties and the silicon concentration in the AlCrSiN coatings has been found and discussed. The coatings formed from cathodes containing less than 5 at% Si crystallize in the cubic structure. The size of the crystallites decreases with the silicon concentration increase. The coatings are characterized by a high hardness with a maximum of about 37 GPa (2 at% Si). The adhesion of the coatings is almost independent of the concentration of silicon. The wear rate is about one order of magnitude higher for coatings deposited from cathodes with a silicon concentration of 5 at% and 10 at% compared to a coating with a lower silicon concentration. This finding is consistent with the results of corrosion resistance studies. The coating deposited from the cathode with 10 at% of silicon exhibits the best anticorrosion properties against the salt solution.

## 1. Introduction

The issue of improving the performance of tools and machine parts is a constant challenge for designers, material experts and technologists. Classic transition metal nitrides such as TiN and CrN, known for a long time, have undergone constant modifications resulting from the challenges of modern industry. The necessary increase in the hardness of protective coatings and their thermal stability, also at high temperatures, forced the design of new, complex, multi-component coatings. Ternary CrXN coatings, where X is metallic (e.g., Ti, Al, Mo, V and Nb); semi-metallic (B and Si) or nonmetallic (O and C) elements [1,2,3,4], are characterized by improved mechanical, tribological and electrochemical properties, as well as their oxidation resistance and thermal stability compared to CrN.

AlCrN is one of more interesting ternary systems. It is characterized by high hardness and, depending on the concentration of aluminum in the coating [1], improved oxidation resistance considerably up to 800 °C [5], better corrosion and wear resistance [1,5,6] compared to CrN. The aluminum concentration plays an important role in the improvement of the properties by affecting the phase structure of the coating. The Al/(Al + Cr) ratio in the coating is a crucial parameter. Dependent on the chemical composition, they can exist with both cubic and hexagonal structures. Systematic studies on the influence of the coating chemical and phase composition on its properties revealed a change of metastable Al_x_Cr_1−x_N solid solution with a face-centered cubic (fcc) structure of B1 type into the B4 hexagonal structure occurring at the Al/(Al + Cr) ratio from 0.6 to 0.75 (dependent on the deposition method) [1,7]. The investigations of Reiter et al. [1] showed that the maximum fraction of AlN in the AlCrN coating forming the cubic phase is 71 at%, although the theoretical predictions indicated even 75 at%.

The hardness of AlCrN ranges from about 32 GPa [5,8] up to 40 GPa [9], depending on the chemical composition of the cathode, the deposition method and the coating deposition conditions. Good properties of AlCrN coatings can be further improved by alloying with elements leading to grain refinement. The introduction of Si into the AlCrN coating results in a further increase in hardness, even to 55 GPa [10]. This is probably due to solid solution [11] and grain boundary [10] hardening phenomena. Due to the grain refinement caused by the silicon addition, the description of the hardness change can be realized using the Hall-Petch relationship. 

Silicon has a very limited solubility in AlCrN; therefore, its excess in the crystal lattice segregates along the grain boundaries. It creates an amorphous a–Si_x_N_y_ phase with embedded MeN crystallites (Me = Al, Cr), i.e., a typical structure of a nanocomposite [12]. With a higher silicon content, a more amorphous phase is formed, and the coatings show a reduced hardness due to grain boundary sliding [10].

Another effect of adding Si to AlCrN is also observed. There is a shift between the cubic and hexagonal crystal structure to lower Al contents. A hexagonal phase is formed for the Al/(Al + Cr) rate characteristic for the cubic phase [11]. A similar effect occurs when AlCrN coatings with Al/(Al + Cr) rate below 0.6 are formed at a high temperature or they are annealed at an elevated temperature [12].

Soldán et al. investigated the properties and structure of the arc-evaporated coatings deposited from the Al_0.70_Cr_0.30_ cathode with Si addition. They found that increasing the concentration of silicon at the expense of chromium in the target increases the Al/Cr ratio in the formed coatings. As a result, in addition to the current phase system (cubic AlN and cubic CrN), an additional phase is created—hexagonal AlN (h–AlN) [13]. The addition of silicon to the AlCrN coating may also affect the nature of the chemical bonding. Metallic bonds dominate in CrN, while, in the AlN phase, as well as SiN, they are characterized by a different type of bond—covalent bonding, stronger than metallic. It may cause changes in the electronic structure and stabilize the hexagonal phase in AlCrN coatings [12].

AlCrSiN coatings are formed by many methods: cathodic arc evaporation [11,12,13,14,15], magnetron sputtering [16,17,18], HiPIMS (High-power impulse magnetron sputtering) [19] or using a hybrid coating system combining a magnetron sputtering (Al and Si cathodes) and arc ion plating (Cr cathode) [10].

The structure [10,11,12,13,14,15,17,18,19], thermal resistance [12] and mechanical [10,11,14,15,16,17,19] and tribological [10,16,17] properties of AlCrSiN coatings of different compositions have been extensively studied. Among the mechanical properties, the hardness and Young’s modulus are the most frequently presented [11,14,16,20]. There is some information on the development of stresses in the coatings with different silicon concentrations [11,19]. However, there are no systematic and detailed studies on the adhesion of the coatings to the substrate. As adhesion is one of the basic factors determining the functional properties of coatings, it seems advisable to take up this topic. Similarly, electrochemical studies are rarely reported in the literature [20,21,22]. Sun et al. [20] investigated AlCrN coatings, obtained by the multi-arc ion plating technique, with the relatively low Al/(Al + Cr) rate range from 0.34 to 0.50 and silicon concentration up to 16 at% and a high concentration of oxygen and carbon up to 5 at%. They found that all coatings showed similar susceptibility to electrochemical corrosion, which was manifested by comparable values of the corrosion potential (about −0.3 V). Further, the coatings wear caused by corrosion decreased slightly with an increasing silicon content.

The paper presents the influence of Si content on the structure, mechanical, tribological and anticorrosive properties of coatings formed from Al_70−x_Cr_30_Si_x_ cathodes with x = 0, 1, 2, 5 and 10 at%, so that, with the increasing silicon concentration, the Al/Cr ratio decreases, contrary to the tests presented by Soldan et al. [13].

## 2. Materials and Methods 

### 2.1. Coating Deposition

Thin AlCrSiN coatings were formed on steel substrates by cathodic arc evaporation. Substrates were made from HS6-5-2 steel (1.3343) with chemical composition (wt.%): C (0.82–0.92), Mn (max 0.40), Si (max. 0.50), P (max. 0.03), S (max. 0.03), Cr (3.5–4.5), W (6–7), Mo (4.5–5.5), V (1.7–2.1), Cu (max. 0.30) and Fe-balanced. They were polished to Ra of about 0.02 µm and washed in an alkaline and ultrasonic bath. After drying, they were mounted in the vacuum chamber of the TINA 900M device (Vakuumtechnik Dresden GmbH, Dresden, Germany) on a rotating table at a distance of 18 cm from the 100-mm in diameter arc sources. For the synthesis of coatings, the following cathodes: Al_70_Cr_30_, Al_69_Cr_30_Si_1_, Al_68_Cr_30_Si_2_, Al_65_Cr_30_Si_5_ and Al_60_Cr_30_Si_10_ were applied. Additionally, the Cr cathode was used to produce the CrN reference coating. Cathode purity, according to the manufacturer certificate, was not less than 99.99%. The chamber was pumped to a pressure of about 1 × 10^−3^ Pa, and then, the substrates were heated to about 350 °C. To remove weakly bound particles and an oxide layer from the substrate, a second stage of substrate cleaning was carried out. It included ion etching with argon and chromium ions under the following conditions: bias of −600 V, argon pressure of 0.5 Pa, current at Cr cathode of 100 A and etching time of 10 min. To improve coating adhesion, a thin layer of chromium (about 0.1 µm) was deposited on the substrate. All coatings were formed under substrate bias voltage of −100 V, nitrogen pressure of 4 Pa and arc current of 100 A. The deposition time was 55 min. The thickness of all coatings was 2.9 ± 0.1 µm. Control of gas flow and pressure in the working chamber was provided by MKS flowmeters (MKS Instruments, Inc., Austin, TX, USA) and Baratron capacitance manometer (MKS Instruments, Inc., Austin, TX, USA), respectively.

### 2.2. Coating Investigations

The program of testing of the deposited coatings included determination of the chemical and phase composition, coating morphology and mechanical and electrochemical properties. The coating thickness was determined using the spherical grinding method. The chemical composition was determined by Jeol JSM 5500 LV (JEOL Ltd., Tokyo, Japan) scanning electron microscope (SEM) equipped with a system for energy-dispersive X-ray spectroscopy (Oxford Link ISIS 300, Link Analytical/Oxford Instruments, High Wycombe, UK). The microscope enabled also studying the surface morphology of the coatings. The Hommel Tester T8000 (Hommelwerke GmbH, Schwenningen, Germany) contact profilograph was used to measure the surface roughness of the coatings.

The grazing incidence X-ray diffraction (Empyrean PANalytical, Malvern Panalytical Ltd., Malvern, UK) was applied to identify the phase composition of the coatings. Cu-Kα radiation (0.154056 nm), the grazing angle ω = 3°, the operating voltage—40 kV, current—35 mA, 2Θ scanning range—20–120°, step size—0.05° and scanning speed—20s/step were applied. HighScore Plus with ICDD PDF 4+ Database software (The Powder Diffraction File^TM^ (PDF^®^) was used for data processing. Based on the diffraction patterns obtained, the crystallite sizes in the coatings were calculated using the Scherrer equation [21]. The Warren and Biscoe correction method, including instrumental peak widening, was also included [23]. For AlCrSiN coatings, the texture coefficient *Tc*(*hkl*) was calculated from the equation [24]:

(1) $$Tc\left(hkl\right)=\frac{I\left(hkl\right)/I_{o}\left(hkl\right)}{\frac{1}{n}\sum_{1}^{n}I\left(hkl\right)/I_{o}\left(hkl\right)},$$

where *I*(*hkl*) and *I_o_*(*hkl*) are the measured and standard diffraction line intensity, respectively, and *n* is a number of analyzed (*hkl*) diffraction lines.

The hardness (H) and Young’s modulus (E) were measured on a Fischerscope HM2000 semi-automatic hardness tester (Fischer Technology Inc., Windsor, CT, USA) equipped with the Bercovich intender, three-sided pyramid with a with tip radius 150 nm and total included angle of 142.3°. In order to correctly determine the hardness of the coatings with high surface roughness, characteristic for the applied technique of their deposition-cathodic arc evaporation, the method described by Romero et al. [9] was used. The coatings were polished using fine alumina powder (1 µm) as the abrasive medium to a roughness of 0.04–0.05 µm. After this operation, the measurement statistics improved significantly. The average hardness value was calculated from at least 20 measurements for three samples. The indenter penetration depth was 0.2 μm. It was less than 0.1 coating thickness. Therefore, the effect of soft substrate, compared to the coating, was avoided. 

To assess the adhesion of AlCrSiN coatings to the steel substrate, results from two research methods were used: the scratch test and the Daimler-Benz test. The Revetest Scratch tester (CSM Instruments, Peseux, Switzerland) was equipped with Rockwell C-type indenter. The test parameters were as follows: scratch length—10 mm, maximum normal load—200 N and scratch speed—10 mm/min. In each measurement (scratch), two critical loads were determined: Lc_1_ at which the process of coating destruction begins and Lc_2_ at which the process of coating detachment is observed. The measurements included at least three scratches, and the critical loads were the average of the individual measurements. The Daimler-Benz test was the second method used to assess adhesion, in which the Rockwell indenter was pressed into the coating with a load of 1471 N. Based on the six-stage scale HF1-HF6, where HF1 means no damage of the coating and only radial cracking and HF6 means extensive coating delamination around the indentation, the coating can be attributed to the appropriate adhesion class. To evaluate the amount and type of coating damage in this test [25], a Nikon Eclipse MA200 microscope (Nikon Corporation, Tokyo, Japan) was applied.

The tribological properties of the coatings, i.e., friction and wear, were determined using a ball-on-disc system, where the tested coating on the steel substrate was a disc, and an Al_2_O_3_ ball with a diameter of 10 mm, a roughness of about 0.03 µm and a hardness of about 16 GPa was a counter-sample. The test parameters were as follows: normal load—20 N, sliding speed—0.2 ms^−1^, sliding radius—11 mm and distance—2000 m. The wear rate was calculated as a volume of the coating material removed during the ball-on-disc test in relation to the product of normal load and distance.

The assessment of the resistance of HS6-5-2 steel covered with AlCrSiN coatings to electrochemical corrosion was done based on the potentiodynamic cathode-anodic polarization method with use of Atlas 0531 (Atlas-Sollich, Rebiechowo/Gdansk, Poland) potentiostat. The tests were carried out in a cell dedicated to electrochemical studies equipped with three electrodes: the reference electrode—a saturated calomel electrode (SCE), the counter electrode—a platinum plate and the working electrode—a separated sample area (about 0.3 cm^2^). A salt solution—3.5 wt% NaCl with the temperature of 25 °C and the pH of 6.5—was used as the electrolyte. 

The so-called potentiodynamic characteristics (polarization curves) were obtained for each of the studied areas. Gaining such characteristics requires a change in potential (*E* and V) of the working electrode over time with simultaneous registration of the current density values (*i* and A/cm^2^) at this electrode. The potential change occurred in the range from a minimum of −0.9 V to a maximum of 1.0 V. In all cases, the rate of potential increase was 0.167 mV/s. The potentiodynamic measurements were carried out after the 5-h stabilization of the samples in the electrolyte, without correcting the potential drop on the solution resistance. During the stabilization, changes in the open circuit potential (OCP) were recorded. Based on the polarization curves obtained (for HS6-5-2 steel uncoated and covered with AlCrSiN coatings), the key electrochemical parameters characterizing the corrosion processes occurring on the surface of the samples were determined using the Tafel extrapolation method [26]. These parameters include corrosion potential (*E*_corr_), corrosion current density (*i*_corr_) and polarization resistance (*R*_p_) at the corrosion potential. The corrosion resistance of the AlCrSiN samples was compared to the properties of a silicon-free AlCrN coating.

The obtained data were subsequently used to estimate the porosity of AlCrSiN (or AlCrN) coatings. For this purpose, the following formula was used [27]:

(2) $$P=\left(\frac{R_{p/s}}{R_{p/c}}\right)\;\times \;10^{-\;\frac{\left|\Delta E_{corr}\right|}{b_{a}}}$$

where *P*—porosity of the AlCrNSi (or AlCrN) coating, *R*_p/s_—polarization resistance of HS6-5-2 steel and polarization resistance of the AlCrSiN (or AlCrN) sample, Δ*E*_corr_—difference between the *E*_corr_ values determined for HS6-5-2 steel and the AlCrSiN (AlCrN) sample and *b*_a_—anodic Tafel slope for HS6-5-2 steel (*b*_a_ = 0.08 V/dec).

Selected areas of the samples, before and after treatment with a corrosive medium (for 5 h under open circuit conditions), were observed using Nikon MA200 optical microscope.

## 3. Results

### 3.1. Surface Morphology

**Figure 1:**
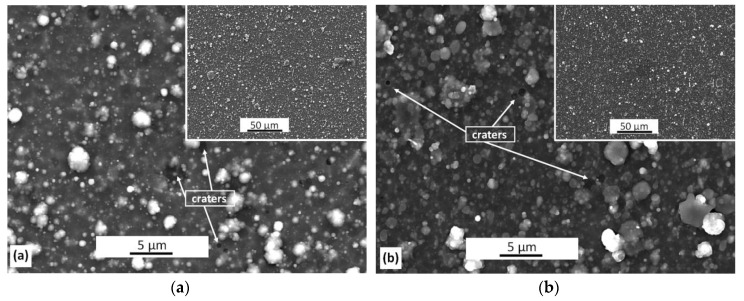
Morphology of: (**a**) AlCrN and (**b**) AlCrSi(10)N coatings.

SEM studies revealed the presence of a large number of macroparticles and craters (white arrows) on the surface of each coating (Figure 1). They are related to the deposition method of the coatings, i.e., cathodic arc evaporation, and are formed as a result of ejection of droplets of the material from high-temperature cathode arc spots [28]. They increase the surface roughness and can deteriorate tribological properties of the coatings. One can observe that the surface images of the surface of AlCrN and AlCrSi(10)N coatings are almost the same, regardless of the cathode used (Figure 1). This proves that the presence of silicon does not significantly affect the surface morphology of the coatings.

The shape of the macroparticles is mainly spherical. Their size ranges from tenths of a micrometer to a few micrometers. About 50–60% of them are characterized by dimensions up to 1 µm, and some reach dimensions even up to 6 µm. A few irregular particles, mainly of larger dimensions, are also visible. Their presence is likely related to the agglomeration of particles ejected from the cathode before reaching the coating surface [29].

**Figure 2:**
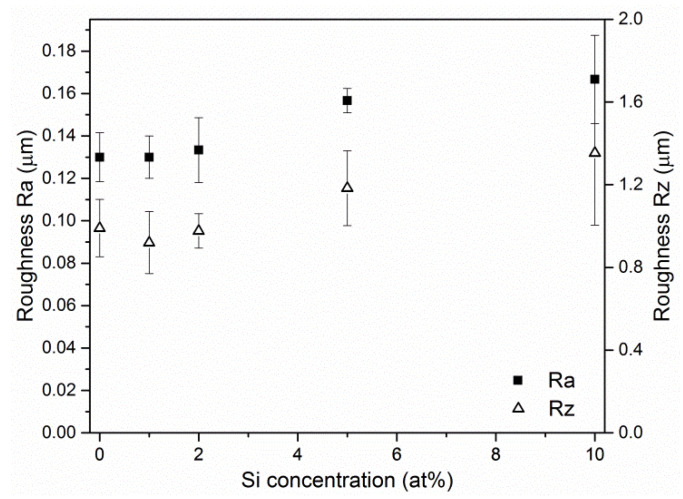
Roughness parameters of the AlCrSiN coatings formed from cathodes with silicon concentrations ranged from 0 at% to 10 at%.

A similar number of surface defects results in a little difference in the surface roughness of the particular coatings (Figure 2). A slight, monotonic increase in the Ra and Rz roughness parameters, from 0.13 µm to 0.16 µm and from 0.99 µm to 1.35 µm, respectively, can be observed. 

Due to the large number of macroparticles of different dimensions on the surface of the coatings, the roughness measurement is burdened with a relatively high measurement uncertainty (Figure 2). These results may indicate a small but measurable dependence of the AlCrSiN coating surface roughness on the silicon concentration. It should be also mentioned that the Ra and Rz roughness parameters for the CrN reference coating are 0.09 µm and 0.76 µm, respectively. Thus, the addition of aluminum into the CrN coating significantly deteriorates its surface quality, causing a Ra and Rz increase by about 30% and 44%, respectively.

### 3.2. Chemical and Phase Composition 

The chemical composition of the coatings is summarized in Table 1. It can be seen that the silicon concentration in the coating is about two times lower than in the cathode. The Al/(Al + Cr) ratio in the coatings is about 0.67 and decreases to about 0.64. It is slightly lower than in the cathode. This is due to the lower atomic mass of Al, which is more dispersed in collisions with nitrogen and leads to lower density in the vapor [30].

**Figure 3:**
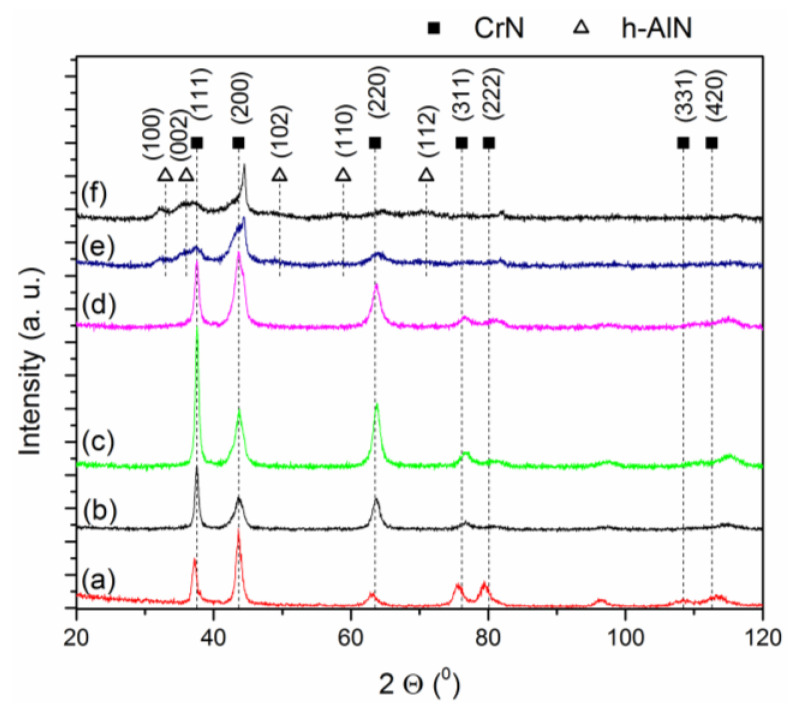
X-ray diffraction (XRD) patterns of CrN (**a**) and AlCrSiN coatings formed from cathodes with silicon concentrations: (**b**) 0 at%, (**c**) 1 at%, (**d**) 2 at%, (**e**) 5 at% and (**f**) 10 at%.

The diffraction lines of CrN and AlCrSiN coatings with different silicon concentrations are summarized in Figure 3. The coatings with silicon concentration up to 2 at% have a B1 cubic structure characteristic for the CrN phase (c-CrN), according to the pattern ICDD 04-004-6868 (Figure 3b–d). The absence of diffraction lines deriving from the AlN phase indicates the total solubility of Al, Cr and N in a NaCl-type microstructure or the coexistence of two AlN and CrN cubic phases crystallizing in the same Fm-3m (225) space group and having lattice parameters of 0.4052 nm and 0.4145 nm, respectively. The silicon-free coating and the coating containing 1 at% of silicon have a strong (111) preferred orientation (Figure 3b,c). Both coatings are highly textured.

According to the standard, the intensity ratio of the diffraction lines 200/111 is 1.22, while, for the tested coatings, is 0.50 (0 at% Si) and 0.41 (1 at% Si in the cathode). The coating formed from the Al_68_Cr_30_Si_2_ cathode exhibits diffraction line intensities similar to the above standard with the 200/111 intensity ratio of about 1.14. The lines are slightly widened, indicating a decrease in the size of the crystallites in the coating. The coatings synthesized from cathodes containing higher silicon concentrations, i.e., 5 at% and 10 at%, show different crystal structures (Figure 3e,f). The intensity of the c-CrN diffraction lines decreases significantly, and the diffraction lines assigned to the hexagonal AlN phase (ICDD 04-012-3388) appear around 33.0° (100) and 36.0° (002). The presence of an intense (101) line located at approximately 37.7° also cannot be excluded. However, it is difficult to clearly confirm due to the proximity of the c-CrN line (111) set at 37.5°. It is worth mentioning that no relevant peaks derived from crystalline silicon nitride were observed in any of the coatings.

In Figure 3a, within the presented range of 2Θ angles, all diffraction lines from the CrN phase are visible, i.e., 111, 200, 220, 311, 222, 400, 331 and 420, with intensities only slightly differing from the standard. The shift of the 111 line due to the presence of aluminum in the CrN network was small, around 0.3°, while, for the 220 line, around 0.7° and, for the 420 line, around 2.0°. The addition of silicon does not result in the diffraction lines shift. This was found by comparison of the XRD pattern for the coating without (Figure 3b) and coating formed from a cathode with a silicon concentration of 2 at% (Figure 3d). The positions of the diffraction lines remain the same.

**Figure 4:**
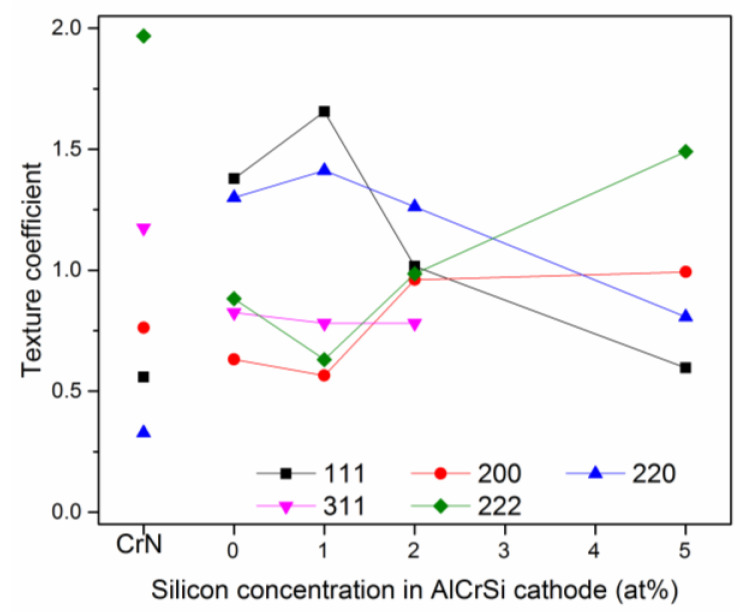
Texture coefficient of the CrN and AlCrSiN coatings formed from Cr cathode and cathodes with silicon concentration ranged from 0 to 5 at%.

The preferred orientation of the crystallites in the coating can be determined by the *Tc*(*hkl*) texture coefficient. It was determined using Equation (1). It defines the degree of preferred orientation of individual planes in the coating. *Tc*(*hkl*) > 1 indicates a preferred orientation when many grains in the coating are arranged in the same way. A *Tc*(*hkl*) close to 1 indicates randomly distributed crystallites. The change of texture of AlCrSiN coatings with various silicon contents and CrN coating as a reference is shown in Figure 4. The most intense diffraction line of the CrN coating (200) has a texture coefficient of about 0.8, while the largest value of the coefficient, about 2.0, refers to the 222 plane. It is likely related to the relatively high intensity of the 111 plane. 

**Figure 5:**
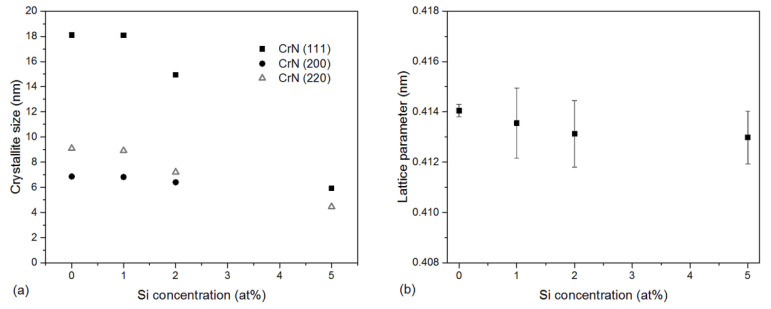
The crystallite size (**a**) and the lattice parameter (**b**) of the AlCrSiN coatings formed from cathodes with silicon concentrations ranged from 0 to 10 at%.

The dashed vertical lines in Figure 3 show the standard position of the diffraction lines. Their analysis showed that, as the silicon concentration in the coating increases, the c-CrN diffraction lines widen. The crystallite sizes in the tested coatings for the CrN (111), CrN (200) and CrN (220) planes are shown in Figure 5a. As can be seen, the silicon concentration in the AlCrSiN coating has a major impact on the size of the crystallites. They are generally small. In the coatings formed from cathodes without silicon or with 1 at% Si, their size is about 18 nm, 7 nm and 9 nm for 111, 200 and 220-oriented crystallites, respectively. There is a visible decrease in the size of crystallites to a value of about 4–6 nm with the increase in silicon concentration in the cathodes.

The observed shift of diffraction lines towards higher angles results in a decrease of the crystal lattice constant from above 0.414 nm (0 at% Si) to below 0.413 nm (5 at% Si) (Figure 5b). A similar effect was observed by Tritremmel et al. [11].

### 3.3. Mechanical Properties

The results obtained during the nanoindentation testing, i.e., microhardness and Young’s modulus of the coatings, are shown in Table 2. Both quantities depend on the silicon concentration. The hardness of the silicon-free coating is about 28 GPa and increases to about 37 GPa (2 at% Si), then decreases to about 24 GPa (10 at% Si). The Young’s modulus shows a similar trend, reaching a maximum value of 358 GPa for the coating formed using the cathode containing 2 at% Si and a minimum equal to 239 GPa (10 at% Si).

According to many authors, these values (H and E) obtained during the indentation test can help to predict the wear of coatings [31,32]. The H/E ratio associated with the elastic strain to failure (plasticity index) can be considered as a factor illustrating the elastic behavior of the coating in contact with the applied load [33]. The H/E values of the tested coatings are close to each other and amount to about 0.1 (Table 2). The smallest value is characteristic for the silicon-free coating, 0.093 ± 0.007, while the highest, 0.112 ± 0.014, for the AlCrSi (1)N coating. 

The resistance to the plastic indentation is described by the H^3^/E^2^ ratio [32]. It is proportional to the load that defines the transition between elastic and plastic contact in a ball-on-plane system. One can state that, as the ratio increases, the coating resistance to plastic deformation increases. The smallest value of this ratio is characteristic for the coating formed from the cathode containing 10 at% Si (0.24 ± 0.06) GPa, while the highest (0.45 ± 0.15) GPa for the coating formed from a cathode with 1% Si (Table 2).

A similar dependence of hardness on the silicon concentration in the coating formed by arc evaporation was observed by Ding et al. [14]. The hardness increased from about 21 GPa (pure CrN) to about 40 GPa when the ratio (Al + Si)/Cr = 1.62. The CrN reference coating obtained by us under the same conditions as AlCrN had a hardness of about 23 GPa, similar to that of Reference [14]. Tritremmel et al. presented a similar trend for AlCrN coatings formed using arc evaporation of the cathode with the chemical composition expressed by the ratio Al/Cr = 1 [11]. The hardness increased from about 26 GPa (Si = 0 at%) to about 36 GPa (Si = 10 at%) and then decreased to about 30 GPa (Si = 20 at%). The deposition method does not seem to have a major impact on this coating property. Coatings obtained by magnetron sputtering have a similar hardness and show a similar nature of the changes [16,17].

Adhesion of the coating to the substrate is one of its most important properties. Among the many methods of adhesion assessment, the scratch test (enabling the numerical representation of the load causing the coating delamination) and the Daimler-Benz test (very fast to perform) are probably the most commonly used.

The adhesion significantly affects the wear resistance of the coatings. In-service damage of the coating, cracking or delamination significantly degrades the anti-wear properties of the coating-substrate system, which can cause severe abrasion to the system. It depends on many factors, including the hardness and the roughness of the substrate [33]. As the hardness increases, the adhesion of the coatings increases. An increase in the roughness of the substrate, in turn, results in an adhesion decrease. To eliminate the effect of these factors on adhesion, the hardness and roughness of the substrates were the same for all samples.

**Figure 6:**
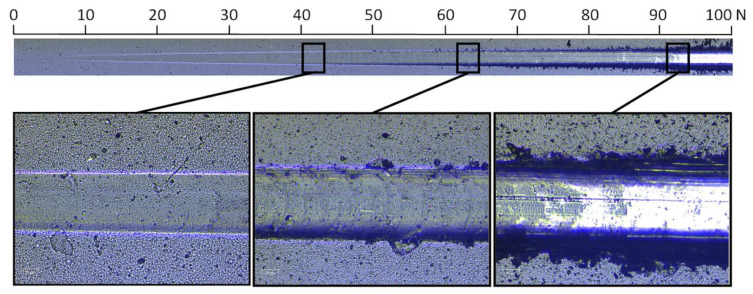
The scratch obtained in a scratch test performed on the coating deposited from the Al_69_Cr_30_Si_01_ cathode and typical scratch adhesion damages at *L*c_1_ = 42 N, *L*c_2_ = 93 N and *L*c = 64 N.

Figure 6 shows the scratch obtained on the AlCrSi(1)N coating and, additionally, a normal load scale. The scratch length is 10 mm, which corresponds to a normal load of 100 N. To make the crack details more visible, its width was doubled. 

The images above, each with widths corresponding to a load difference of 3 N, show the enlarged details of the scratch for the characteristic loads: Lc_1_—cohesive failure at the appearance of the first crack (*L*c_1_ = 42 N) and *L*c_2_—adhesive failure corresponding to the complete removal of the coating from the substrate (*L*c_2_ = 93 N). The image of the crack for an intermediate load (*L*c = 64 N), during which the cracks propagate under load, is also presented. At this load, a slight flaking of the coating on a border of the scratch can be observed. It is worth noting that the average thickness of the coatings is about 3 µm, and the bottom of the scratch under the load of Lc_1_, intermediate load and Lc_2_ are at the depths of 11 µm, 18 µm and 29 µm, respectively. Due to the large difference in the hardness of the coating and the substrate, different processes occur at the above loads (and corresponding depths of scratch bottoms). At Lc_1_, a slight plastic deformation of the substrate does not cause cracking of the coating; however, at greater depths, cracking (flaking) at the scratch border can be observed.

The critical loads for the coatings with different silicon concentration Lc_2_ ranges from about 90 N to about 100 N (Table 2). There is no significant change in the critical load with the change of silicon concentration in the coatings. It seems that, for higher silicon concentrations, the critical load may be slightly lower than for coatings with a low silicon concentration. CrN and AlCrN coatings are characterized by very good adhesion, with the critical force Lc exceeding 80 N [3,34].

**Figure 7:**
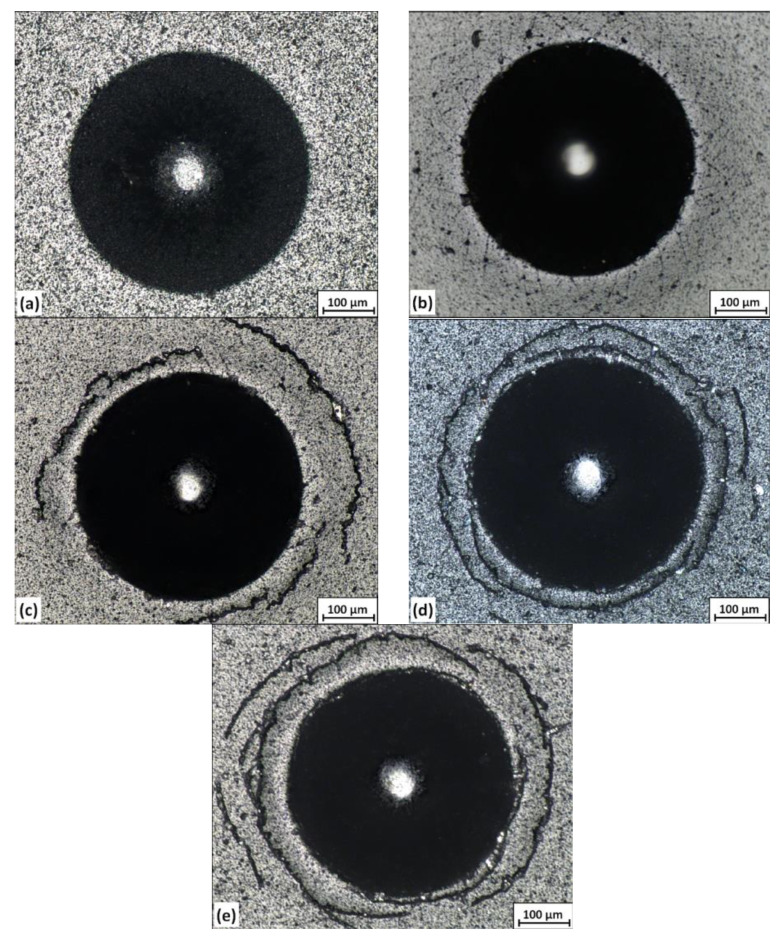
The images of indentations in the Daimler-Benz test of AlCrSiN coatings formed from cathodes with silicon concentrations: (**a**) 0 at%, (**b**) 1 at%, (**c**) 2 at%, (**d**) 5 at% and (**e**) 10 at%.

The AlCrN coating shows no significant cracks around the indentation in the Daimler-Benz test (Figure 7a). The coating can be evaluated to adhesive strength HF1, which indicates a strongly adherent coating. There are numerous, short radial and oblique cracks around the indentation in the coating formed from the cathode containing 1 atm.% Si (Figure 7b). Due to the greater number of cracks, the adhesive strength can be classified as HF2. Only slight radial cracks occur in coatings formed from cathodes containing 2, 5 and 10 at% Si (Figure 7c–e). However, circular cracks are visible, and their number increases with the silicon concentration. Due to the small delamination at the edge of the indentation or at circular cracks, the adhesive strength can be classified as HF3. It should be stated that there is no massive delamination around the indentations in the tested coatings, as it would disqualify the coating from industrial applications.

### 3.4. Tribological Properties

**Figure 8:**
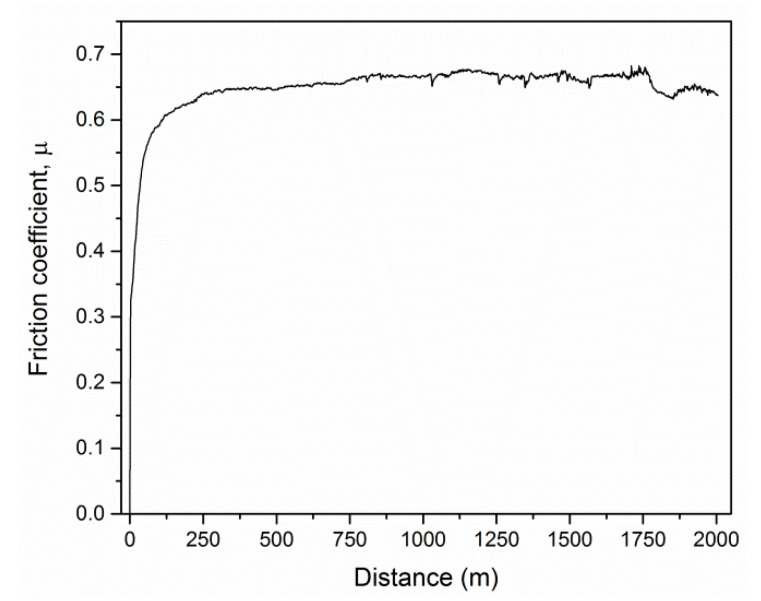
Friction behavior of the AlCrSiN coating deposited from the cathode with a silicon concentration of 1 at%.

It is known that the type of counterpart, as well as the parameters of the friction test, determine the friction coefficient and wear [8,35]. The possibility of creating bonds between the coating and the counterpart increases the interaction between them, causing an increase in the friction force. For this reason, all coatings were tested in the same ball-on-disc system and conditions. Figure 8 shows the behavior of the friction coefficient versus the distance for a selected coating formed from the Al_69_Cr_30_Si_1_ cathode.

The tests carried out against the Al_2_O_3_ counterpart showed that the friction coefficient of the coatings fluctuated without a clear trend and ranged from 0.62 to 0.68. The changes in the friction coefficient during the test proceeded similarly for all coatings. During the running-in period (distance of about 300 m), a steady increase in the friction coefficient to the value of about 0.65 was observed. After the running-in period, the coefficient value was constant, with slight fluctuations, especially at the end of the test. No sudden changes in the friction coefficient were observed that would suggest a change in the friction environment.

**Figure 9:**
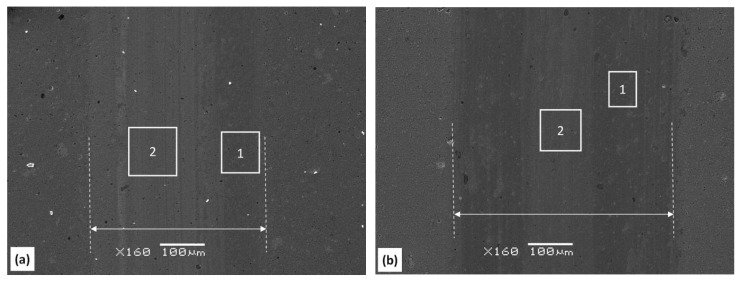
SEM micrographs and chemical composition from energy-dispersive X-ray spectroscopy (EDX) of the wear tracks obtained after the ball-on-disc test at AlCrSiN formed from cathodes with silicon concentrations of (**a**) 1 at% and (**b**) 10 at%. The rectangles indicate the areas of EDX analyses.

Figure 9 shows SEM micrographs of the wear tracks of AlCrSi (1)N and AlCrSi(10)N coatings after the above-mentioned test. The substrate material was not identified within the wear tracks, indicating that the coatings were not completely worn. 

The wear depth after about 29,000 revolutions, in the friction test, was about 0.22 µm and about 0.65 µm for AlCrSi (1)N and AlCrSi (10)N, respectively. A greater wear depth translates directly to a greater wear width. The boundaries of the wear tracks are marked with white broken vertical lines. The wear width is about 380 µm and 500 µm for the coatings with lower and higher silicon concentrations, respectively.

**Figure 10:**
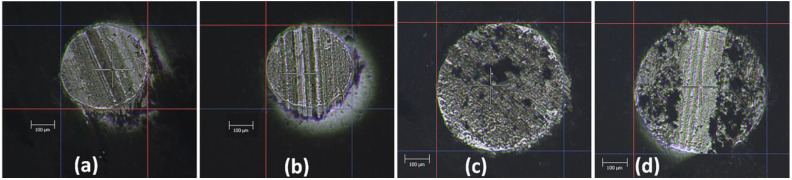
Wear of the counterpart, Al_2_O_3_ ball, after the test with the coatings deposited from cathodes with silicon concentrations: (**a**) 1 at%, (**b**) 2 at%, (**c**) 5 at% and (**d**) 10 at%.

This trend is confirmed in Figure 10, which shows the wear of some Al_2_O_3_ counterparts after the friction test. There is a direct relation with Table 2 here; the lower the wear rate, the smaller the abrasion diameter of the counterparts.

**Figure 11:**
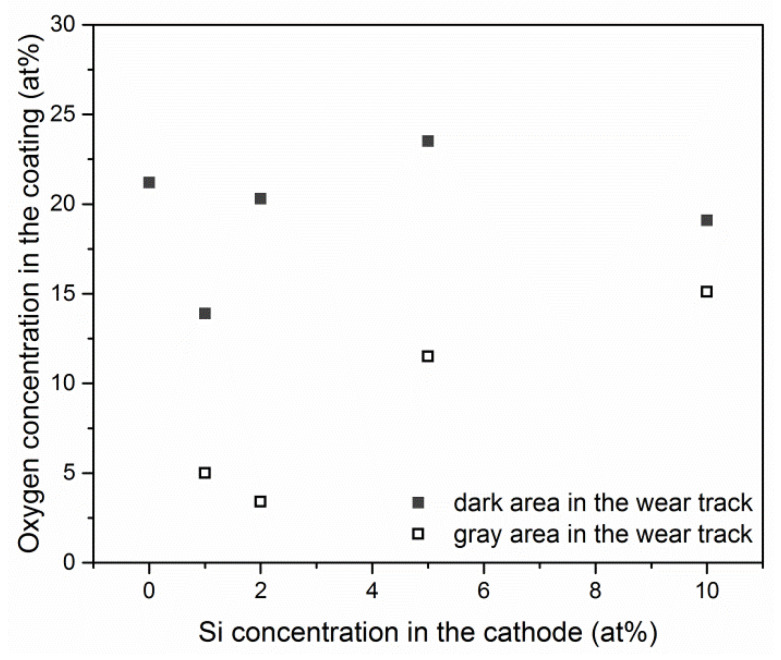
Oxygen concentrations in the dark and gray areas of AlCrSiN coatings deposited from cathodes with various silicon concentrations.

The wear tracks were examined for tribochemical reactions that could occur during the friction test. These were analyzed by EDX at various points of the wear tracks and coatings. Figure 9 shows the areas within the friction tracks, marked with numbers 1 and 2, where the chemical composition analysis was carried out. It was found that there is a much higher oxygen concentration in so-called dark areas (symbol 1) than in gray areas (symbol 2) (Figure 11). Simultaneously, the Al/(Al + Cr) ratio in the wear tracks of the coatings (Table 1) remains similar. At points outside the wear tracks, the oxygen content ranged from 0.7 to 1.6 at%, i.e., it was close to the values presented in Table 1.

The friction coefficient of the AlCrSiN coatings tested against the WC counterpart ranges from about 0.2 [16,20] to about 0.8 [2,16]. The differences may result from the application of different sliding speeds and normal loads during the tests. In the case of the Al_2_O_3_ counterpart, the friction coefficient is about 0.6 regardless of the silicon concentration in the coating [16] or even lower, about 0.55 [36]. 

The wear tracks are wide and smooth, without traces of any components—microchips or delaminated coating parts. No damage (cracks and delamination) of the coatings is observed there. The analysis of the wear tracks did not reveal the presence of the substrate dominant element—iron. Only the coating elements and an increased amount of oxygen were observed. It seems that only the abrasive wear of the coatings is observed in a tribological contact.

The wear track widths shown in Figure 9a,b are about 380 nm and about 500 nm, respectively, while the wear diameters of the counterparts working with these coatings are about 390 µm and about 510 µm, respectively. The normal load, sliding distance and speed, as well as the circular track of a 22-mm diameter against the Al_2_O_3_ counterpart in the ball-on-disc test, were the same.

### 3.5. Electrochemical Properties

**Figure 12:**
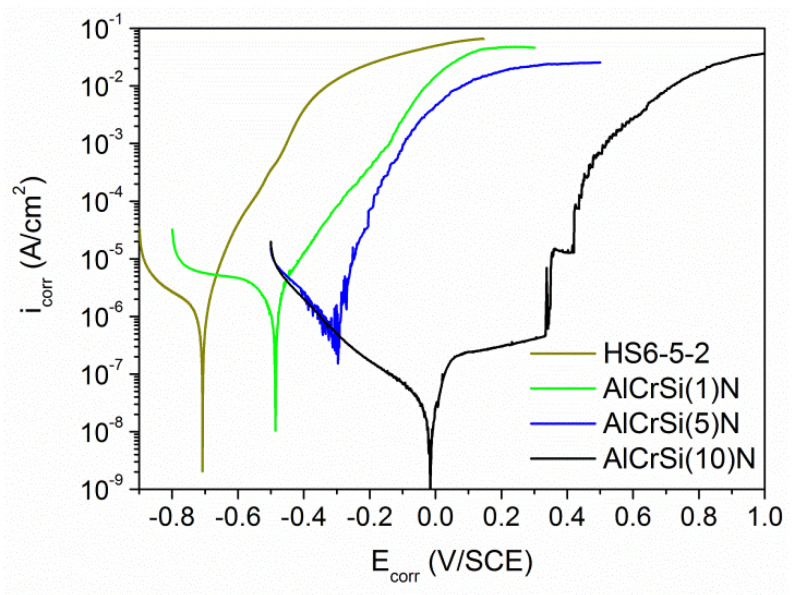
Results of the potentiodynamic polarization tests obtained for the HS6-5-2 and HS6-5-2/AlCrSiN systems.

Figure 12 presents the current-voltage characteristics (polarization curves) obtained for the coatings deposited onto HS6-5-2 steel from cathodes with different silicon contents varying from 1 at% to 10 at%. The calculated corrosion parameters (*E*_corr_, *i*_corr_ and *R*_p_), including the porosity of the coatings (*P*), are gathered in Table 3. In addition, Figure 12 shows the polarization curve obtained for the pristine substrate. This allows to demonstrate that all AlCrSiN coatings reduce the susceptibility of steel to electrochemical corrosion in the saline solution used. The above statement is confirmed by the *E*_corr_ values estimated for HS6-5-2/AlCrSiN systems that are higher compared to HS6-5-2 in each case (see Table 3).

Simultaneously, a gradual shift of *E*_corr_ towards more noble potential values is observed with the increasing Si content. The highest value of the corrosion potential was recorded for the AlCrSi (10)N coating. The shape of the polarization curve obtained in this case differs notably from the current-voltage characteristic received for the substrate. The specimen shows clear active-passive behavior in the measurement environment applied. This results in the occurrence of three characteristic ranges along the anodic branch. They correspond to active dissolution of the coating components ranging from *E*_corr_ to about 0.036 V, an active-passive transition and an unsteady passive range observed until the breakdown potential (*E*_b_) is reached at the potential value of 0.335 V.

In contrast to AlCrSi(10)N, the remaining AlCrSiN samples exhibit a similar course of the anodic polarization curves to that obtained for the steel substrate. They are characterized by a high increase of the anodic current density in a narrow potential range. Thus, although the AlCrSi(1)N and AlCrSi(5)N coatings show higher corrosion potentials compared to HS6-5-2, beyond the *E*_corr_ values, oxidation of the substrate components occurs at a high rate, as evidenced by the *i*_corr_ and *R*_p_ determined for these specimens. 

**Figure 13:**
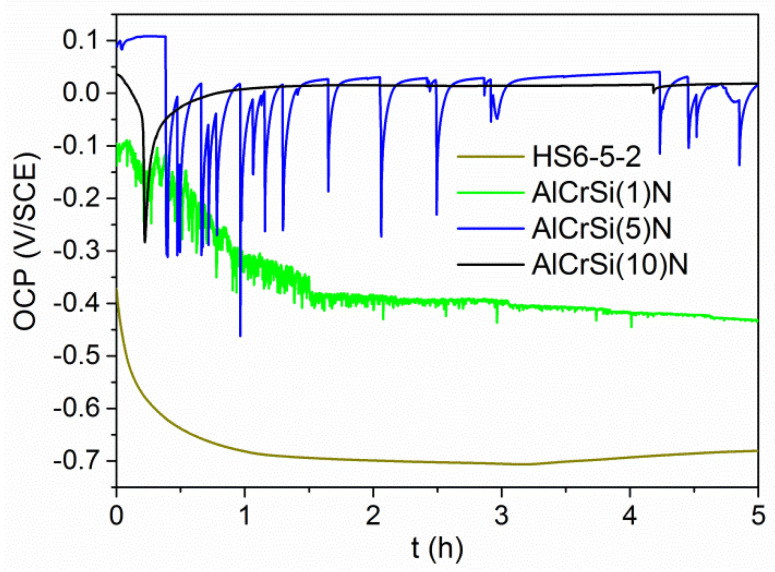
Open circuit potential (OCP) changes monitored during the five-hour stabilization of the HS6-5-2 and HS6-5-2/AlCrSiN systems in the electrolyte solution

**Figure 14:**
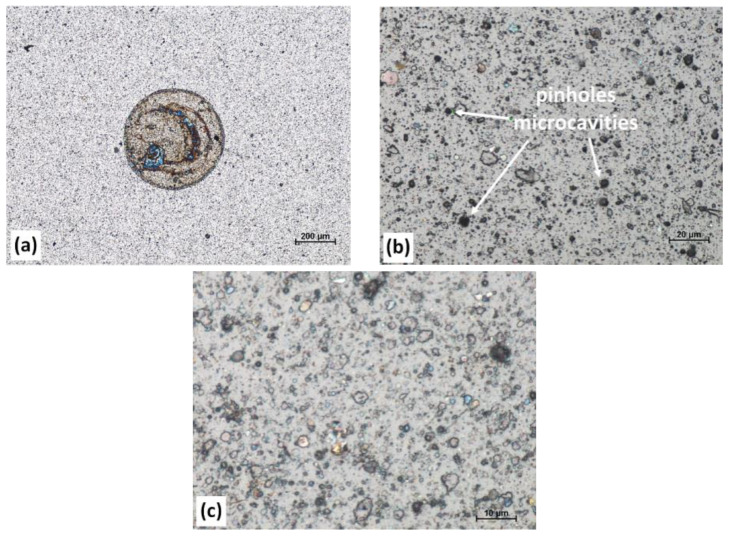
Microscopic (optical) images recorded after five-hour immersion of: (**a**) AlCrSi(1)N, (**b**) AlCrSi(5)N and (**c**) AlCrSi(10)N in the 3.5 wt% NaCl solution.

The curves representing the OCP changes in time (Figure 13) and the microscopic images recorded after five-hour immersion of the AlCrSiN samples in 3.5 wt% NaCl solution (Figure 14) are consistent with the observations made during the potentiodynamic measurements. 

For the respective samples, it was found:

- AlCrSi (1)N—a decrease in OCP, gradually tending to reach a value close to the corrosion potential of HS6-5-2 steel. This inclination indicates the deposit discontinuity. The porosity of this coating reached the highest value of 0.2 % (see Table 3). The optical image (Figure 14a) reveals extensive corrosion centers occurring in the vicinity of defects in the AlCrSi (1)N coating.
- AlCrSi(5)N—strong fluctuations of OCP likely resulting from the contact of the electrolyte with the adhesive layer via the pinholes/microcavities, visible in Figure 14b, and/or the transpassivation and passivation processes taking place at the surface of the coating.
- AlCrSi(10)N—stable OCP over a wide time interval, indicating high chemical inertness of the coating in the corrosion environment used. The optical image of the tested sample area showed no significant damages to the AlCrSi (10)N coating (Figure 14c).

**Figure 15:**
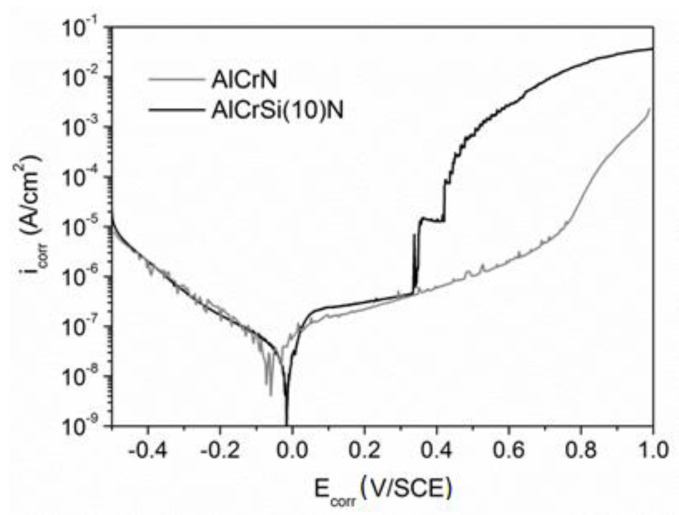
Results of the potentiodynamic polarization tests obtained for the HS6-5-2/AlCrSi(10)N and HS6-5-2/AlCrN systems.

The properties of AlCrSi (10)N were further compared with the reference HS6-5-2/AlCrN system (the sample labeled as AlCrN) equipped with a silicon-free coating (Figure 15). It should be noted that fluctuations observed in the course of the polarization curves, particularly in the case of AlCrN, resulted from low current values measured at the sensitivity limit of the device—the potentiostat. Both specimens were found to show favorable electrochemical parameters and high tightness, i.e., their porosity can be considered negligible (Table 3). The microscopic image recorded for the AlCrN area treated with the electrolyte (not shown here) revealed a lack of significant coating defects, similarly to AlCrSi (10)N. However, no breakdown potential was found in the course of the polarization curve obtained for the AlCrN sample; the coating gradually dissolved at a low rate. 

## 4. Discussion

As shown in Table 1, the Al/(Al + Cr) rate in the coatings analyzed in this work is relatively close to the critical values (even 0.71 [1]) and decreases from 0.67 to 0.64 with the increasing silicon concentration in the cathode. Although the coatings formed from cathodes containing up to 2 at% of silicon are characterized by the highest Al rate (0.65–0.67), a coating is formed in a purely cubic CrN phase (Figure 3b–d). A higher silicon concentration in the cathode (5 at% and 10 at%) causes a decrease in the Al/(Al + Cr) rate but, also, the formation of the hexagonal AlN phase (Figure 3e,f). This can be attributed to the presence of silicon in the coating. As a consequence, this can lead to changes in the microstructure, including the size of the crystallites in the coating.

The hexagonal phase in AlTiN and AlCrN may be also formed as a result of significant compressive stresses or vacancies [12]. The investigations of Hans et al. [37] of AlTiN coatings indicated that also the surface energy and, thus, the size of crystallites may determine the formation of phases of metastable materials. The calculations of the stability range of the cubic and hexagonal phases in comparison to the size of the crystallites indicate that the formation of the hexagonal phase is favored for the coatings characterized by fine crystallites. These studies confirm the previous conclusions about limiting the growth of crystallites by the amorphous SiNx phase segregating at their boundary and destabilizing the cubic AlCrN phase [38].

Many authors indicated that even a small addition of another element to the coating significantly affects the size of the crystallites. Nose et al. found that an increase in the concentration of boron in the AlCrN coating causes a reduction of crystallites oriented in the (111) plane from about 23 nm to about 10 nm and in the (200) plane from about 12 nm to about 5 nm [39]. Tritremmel et al. confirmed the decrease in the crystallite size from about 35 nm to about 6 nm in AlCrSiN coatings produced by cathodic arc evaporation from AlCrSi cathodes, in which Al/Cr = 1 and the silicon concentrations from 0 to 20 at% [11].

Coatings formed without or with a little addition of silicon have a B1-type cubic structure with diffraction lines corresponding to the CrN phase (Figure 3). Coatings with a higher silicon concentration (5 and 10 at% in the cathode) are characterized by a two-phase structure. They constitute a mixture of the cubic CrN phase and the hexagonal AlN phase (Figure 3e,f). Aluminum atoms with a smaller atomic radius (125 pm) substitute chromium atoms with a larger radius—140 pm. It should be assumed that, despite exceeding the solubility limit of Al in the cubic structure of CrN (66 at% [1]), there exists a metastable supersaturated fcc AlCrN solid solution. Investigations of coatings produced by the magnetron sputtering method from the AlCr alloy cathode (70:30) confirm the absence of the developed hexagonal AlN phase in the AlCrN coating [40]. Additionally, substituting Si atoms to the CrN lattice causes its distortion due to differences in the sizes of the atoms.

Silicon in AlCrN solid solution can occupy interstitial positions or substitute Al and/or Cr atoms. The radius of the Si atoms is 110 pm and is smaller compared to Al and Cr. Therefore, it is expected that the AlCrSiN solid solution will exhibit diffraction lines shifted towards higher 2Θ angles. This results in a reduction of the lattice parameter, as shown in Figure 4. Tritremmel et al. found that the solubility of silicon in the fcc AlCrN lattice is limited. Si atoms segregate at the grain boundaries, creating an amorphous SiNx phase there, which results in a reduction in grain size [11].

It can be also noticed that, by increasing the amount of Si in the coating, the intensity of the (111) CrN diffraction line clearly decreases, and the intensity of the (200) CrN line increases. The ratio of the intensity of these I_(111)_/I_(200)_ lines is about 1 for the coating formed from the cathode with a silicon concentration of 2 at%. The studies of Chen et al. [41] indicated that changes in the orientation of fcc coatings can be interpreted on the basis of surface energy and deformation. The fcc coatings tend to grow along the direction with less strain energy, generally 111. With the formation of amorphous silicon nitride at the CrN grain boundary, the crystallite growth in the preferred (111) orientation is limited. As the silicon concentration is further increased in the coating, the (111) CrN intensity disappears. The crystallites grow along the (200) direction, with the lowest surface energy. They form a fine-grained structure in CrAlSixN coatings thanks to the inclusion of the amorphous Si_3_N_4_ phase, and therefore, the width of the diffraction lines increases [41].

The change in the crystallite size observed in Figure 5 and the change in the texture of the coatings (Figure 4) may affect their mechanical properties. The AlCrN coating, without the addition of silicon, has a hardness of about 28 GPa. The addition of silicon, which can occupy interstitial sites or substitute for Al or Cr, affects the deformation in the coating by inducing a local stress field. A similar effect was observed by Tritremmel et al. [11]. They also showed the similarity of changes in residual compressive stresses and hardness for low silicon concentrations, from which they concluded about the inclusion of silicon in the fcc AlCrN phase. Many authors also confirm the formation of the amorphous SiNx phase at the grain boundaries being a barrier to the dislocation movement [42]. This causes grain size refinement and, in accordance with the Hall-Petch rule, an increase in the hardness of the coating [43]. As the silicon concentration increases (5 and 10 at%), the volume of the amorphous SiNx phase also increases. The intensity of the fcc CrN diffraction lines (Figure 3) significantly decreases or even disappears. This is due to the reduction of the crystallite size to about 5 to 6 nm (Figure 5). Additionally, the AlN hexagonal phase with low hardness is formed. Both of these factors can reduce the coating hardness.

The H/E ratio can define the toughness of the coatings and their resistance to abrasive wear or cracking. H/E = 0.1 separates the two zones: the plastic (below) and elastic zones (above) [44]. Coatings with H/E > 0.1 should have improved wear resistance. This means that all coatings should show good wear resistance. The H^3^/E^2^ ratio enables the prediction of their wear resistance. The smallest value is for the AlCrSi(10)N coating (0.24 ± 0.06) GPa, while the highest (0.45 ± 0.15) GPa is for the AlCrSi(1)N coating. It strongly corresponds to the wear resistance (Table 2). Additionally, a lower H^3^/E^2^ ratio for coatings with a higher concentration of silicon, indicating their worse wear resistance, may also be an indicator of lower adherence of the coatings to the substrate.

The investigations of Li et al. [19] showed that the hardness decreased with the increase of silicon concentration in the coating, which was confirmed by the results presented earlier [11] and in this paper, but the compressive stress also increased [11]. The stress increase may reduce the adhesion of the coatings. Tritremmel et al. indicated that the stress value depends on the substrate bias voltage applied during the formation of the coating, and the stress increased with the increasing bias [11]. Such a high value of the critical load indicates that the coatings are difficult to detach from the substrate. At a relatively high nitrogen pressure during the coating deposition, p_N2_ = 4 Pa, there are many random collisions, which reduces the kinetic energy of ions. Defects are often the source of crack initiation and coating destruction. However, despite the large number of surface defects, macroparticles and craters (Figure 1), the coatings are characterized by a high critical load.

The high load applied in the Daimler-Benz test generates high tensile stresses in the vicinity of the indentation. As the indenter is pressed into the coating (and the substrate), most of the kinetic energy is converted into a plastic work of penetration. Some of the substrate material changes position and flows out around the indent. This causes in-plane tensile stresses, which may result in cracking of the coating [40]. In the case of hard, brittle materials, these stresses result in the formation of large round cracks in the peripheral region [45,46].

The deterioration of the wear resistance of the AlCrSi(5)N and AlCrSi(10) coatings may be associated with the softer and rougher nature of the coatings. The Al_2_O_3_ counterpart wear rate is approximately one to two orders of magnitude lower than that of the tested coatings. The wear diameter of the counterparts (Figure 10) correlates with the width of the coating wear tracks. Sun et al. found a five-fold increase in the wear rate with a silicon concentration increase from 2.9 at% (4 × 10^−7^ mm^3^/Nm) to 15.6 at% (about 8 × 10^−6^ mm^3^/Nm) [20]. 

As shown in Figure 11, a higher oxygen concentration is observed in the so-called dark and gray areas in the wear tracks (Figure 9) than outside the friction trace. This is probably related to the adsorption of oxygen in the friction track from the surroundings. The applied parameters of the friction test, i.e., the normal load of 20 N and the sliding speed of 0.2 m/s, favor the occurrence of the so-called flash temperature [8,47]. It can even exceed 1000 °C [8]. This facilitates the formation of Al_2_O_3_ and Cr_2_O_3_ oxides constituting the tribolayer. There is a greater amount of oxygen, regardless of the silicon concentration in the coating, in the dark areas (marked as 1 in Figure 9) compared to the gray ones (marked as 2 in Figure 9). Lin et al. [48] indicated that this may be related to a higher local temperature during sliding, which promotes the formation of the oxide phase in the friction track. Additionally, numerous tests of the top layer of the coating using the XPS method indicate an increased amount of oxygen in this part of the coating compared to the interior [5,20,35]. The difference in the colors of the coating may be related to the thickness of the oxide layer.

The oxide layer formed in the friction track has different mechanical and thermal properties than the coating, so stresses are expected to occur between them. This layer can crack under certain conditions, and loose fragments can act as a so-called third body, increasing the wear of the coating. A higher oxygen concentration on the surface of friction tracks of coatings formed from cathodes with a higher silicon concentration promotes the formation of greater amounts of oxides and a greater amount of debris due to their possible cracking, increasing the wear of the coatings.

It was observed that the silicon concentration influences the corrosion behavior of the AlCrSiN coatings. On the basis of the electrochemical tests performed, the AlCrSi(10)N coating was selected as a good anticorrosion protection for HS6-5-2 steel in the environment of a 3.5 wt.% NaCl solution. The polarization curve obtained for the sample (Figure 12) showed the presence of a passive range manifested by a decrease in the anodic current accretion rate. Such a result indicated the formation of a passive layer at the AlCrSi(10)N surface, blocking the access of the electrolyte to the reactive components of the coating/substrate. The presence of a densely structured layer, sealing the coating surface, was confirmed by the values of other electrochemical parameters i.e., low *i*_corr_, high *R*_p_ and minimal coating porosity equaling to 5.5 × 10^−9^% (see Table 3). Therefore, one can conclude that the corrosion processes observed until the breakdown potential value occur only within the AlCrSi(10)N coating, without the substrate involvement. These favorable features are likely related to the significant share of Si in the coating composition. Silicon, by forming SiO_2_ (particularly in an oxidizing environment) and by contributing to the structure amorphization, tends to stabilize the passive state of the deposit [20,49]. The above statements are supported by the phase composition studies of the AlCrSi(10)N coating. They showed the presence of a considerable volume of the SiN_X_ phase segregated at the grain boundaries and contributing to the grain size refinement. The breakdown of passive layer (at *E*_b_) and/or the AlCrSi(10)N coating fracture is presumably caused by internal compressive stresses [50], which are greater than for the silicone-free coating [11].

The remaining AlCrSiN coatings, after exceeding the *E*_corr_ values (Figure 12), undergo oxidation according to the mechanism of homogeneous corrosion representative for HS6-5-2 steel [51]. This indicates the participation of the substrate in the corrosion processes observed. The probable cause is a relatively high porosity of the coatings—0.2% and 1.5 × 10^−3^ % for AlCrSi(1)N and AlCrSi(5)N, respectively. It should be noted that the porosity determined by comparing the polarization resistances of the coating and the uncoated substrate applies to the open porosity only. Coating discontinuities, e.g., pinholes, microcracks and gaps formed at microdroplets, facilitate the electrolyte to contact the substrate. In the microcells created between the coating and the substrate, steel adopts the anode function and undergoes dissolution [52]. Such defects are typical for the coatings obtained by the cathodic arc evaporation. However, in the case of high-hardness coatings, as AlCrSi(1)N, microcracks may also appear under measurement conditions as a correlated effect of cyclic stress (caused by the pressure of an electrochemical cell equipped with a Teflon O-ring seal) and water-based erosion (refer to Figure 14a). In addition, a low Si content reduces the “sealing” effect observed for AlCrSi(10)N. Both these effects contribute to the high value of the AlCrSi(1)N coating porosity, determined in electrochemical measurements.

## 5. Conclusions

A set of coatings with different silicon concentrations was synthesized using cathodic arc evaporation. The conclusions are summarized as follows:

1. The addition of silicon to AlCrN results in phase composition modification. The preferred crystalline orientation from (111) to (200) plane and the transformation from cubic AlN to a hexagonal one is observed. Increase in the silicon concentration results in crystallite size and lattice parameter decreases.
2. All the coatings exhibit high hardness, with a maximum of about 37 GPa for coatings formed from the Al_68_Cr_30_Si_2_ cathode. The critical load corresponding to the delamination of the coating is similar for all coatings and exceeds 90 N. The silicon-free coating has the lowest amount of damage around the indentation in the Daimler-Benz test and exhibits adhesive strength HF1. The addition of silicon into the coating deteriorates its adhesion to the substrate, the amount of damages around the indentations increases and, due to the small coating delaminations, they are characterized by the adhesive strength of HF2 and HF3.
3. The friction coefficient of the coatings investigated is almost independent of the silicon concentration. The coatings formed using cathodes with higher silicon concentration, i.e., 5 and 10 at% show about one level of magnitude higher wear rate compared to other coatings.
4. All AlCrSiN coatings investigated increased the corrosion resistance of HS6-5-2 steel in salt solutions. The coating deposited from the Al_0.60_Cr_0.30_Si_0.10_ cathode showed the best anticorrosion properties. The electrochemical parameters determined for the AlCrSi(10)N coating are comparable to those of AlCrN, i.e., the coating obtained under similar conditions from the Al_70_C_.30_ cathode.

## Figures and Tables

**Table 1:** The chemical composition of the coatings deposited from different cathodes.

Cathode	Coating Composition (at%)					Al/(Al + Cr) Rate		Coating Designation
	Al	Cr	Si	O	N	Cathode	Coating	
Al_70_Cr_30_	38.2	18.4	0.0	0.6	43.4	0.7	0.675	AlCrN
Al_69_Cr_30_Si_1_	34.7	17.5	0.4	0.7	47.4	0.697	0.665	AlCrSi(1)N
Al_68_Cr_30_Si_2_	33.9	18.1	1.1	0.9	46.9	0.694	0.652	AlCrSi(2)N
Al_65_Cr_30_Si_5_	33.4	18.3	2.3	0.7	46.0	0.684	0.646	AlCrSi(5)N
Al_60_Cr_30_Si_10_	31.1	17.3	4.4	0.8	47.2	0.667	0.643	AlCrSi(10)N

**Table 2:** Mechanical and tribological properties of the AlCrSiN coatings.

Parameter	AlCrN	AlCrSi (1)N	AlCrSi (2)N	AlCrSi (5)N	AlCrSi (10)N
Hardness, H (GPa)	28.5 ± 1.3	29.8 ± 3.0	36.6 ± 3.3	28.8 ± 1.9	24.1 ± 1.2
Young’s Modulus, E (GPa)	306 ± 10	327 ± 14	358 ± 24	261 ± 14	239 ± 10
H/E	0.093 ± 0.007	0.112 ± 0.014	0.102 ± 0.016	0.110 ± 0.013	0.101 ± 0.009
H^3^/E^2^	0.25 ± 0.05	0.45 ± 0.15	0.38 ± 0.15	0.35 ± 0.11	0.24 ± 0.06
Lc_2_ (N)	97.2 ± 0.9	96.0 ± 3.6	98.5 ± 2.0	87.7 ± 0.8	98.1 ± 2.5
Wear Rate (mm^3^/Nm)	(2.4 ± 1.1) × 10^−8^	(3.8 ± 1.9) × 10^−8^	(4.3 ± 0.9) × 10^−8^	(2.9 ± 0.6) × 10^−7^	(2.7 ± 0.2) × 10^−7^

**Table 3:** Electrochemical parameters estimated for the HS6-5-2 and HS6-5-2/AlCrSiN systems.

Parameter	HS6-5-2	HS6-5-2/AlCrSiN (AlCrN)			
		AlCrN	AlCrSi (1)N	AlCrSi (5)N	AlCrSi (10)N
*E*_corr_ (V)	−0.702	−0.075	−0.491	−0.293	−0.014
*i_corr_* (A/cm^2^)	2.8 × 10^−6^	3.7 × 10^−7^	3.1 × 10^−6^	6.4 × 10^−6^	1.0 × 10^−7^
*R*_p_ (Ωcm^2^)	1.3 × 10^4^	1.3 × 10^6^	1.4 × 10^4^	6.8 × 10^3^	0.6 × 10^6^
*P* (%)	–	1.5 × 10^−8^	0.2	1.5 × 10^−3^	5.5 × 10^−9^
